# Chronic Hypergravity Induces a Modification of Histone H3 Lysine 27 Trimethylation at TCRβ Locus in Murine Thymocytes

**DOI:** 10.3390/ijms23137133

**Published:** 2022-06-27

**Authors:** Gaetano Calcagno, Nassima Ouzren, Sandra Kaminski, Stéphanie Ghislin, Jean-Pol Frippiat

**Affiliations:** Stress, Immunity, Pathogens Laboratory, SIMPA, Université de Lorraine, F-54000 Nancy, France; gaetano.calcagno@univ-lorraine.fr (G.C.); nassima.ouzren-zarhloul@univ-lorraine.fr (N.O.); sandra.kaminski@univ-lorraine.fr (S.K.)

**Keywords:** hypergravity, stress, T lymphopoiesis, V(D)J recombination, epigenetic, EZH2

## Abstract

Gravity changes are major stressors encountered during spaceflight that affect the immune system. We previously evidenced that hypergravity exposure during gestation affects the TCRβ repertoire of newborn pups. To identify the mechanisms underlying this observation, we studied post-translational histone modifications. We first showed that among the four studied post-translational histone H3 modifications, only lysine 27 trimethylation (H3K27me3) is downregulated in the thymus of mice exposed to 2× *g* for 21 days. We then asked whether the TCRβ locus chromatin structure is altered by hypergravity exposure. ChIP studies performed on four Vβ segments of the murine double-negative SCIET27 thymic cell line, which corresponds to the last maturation stage before V(D)J recombination, revealed increases in H3K27me3 after 2× *g* exposure. Finally, we evaluated the implication for the EZH2 methyltransferase in the regulation of the H3K27me3 level at these Vβ segments by treating SCIET27 cells with the GSK126-specific inhibitor. These experiments showed that the downregulation of H3K27me3 contributes to the regulation of the Vβ germline transcript expression that precedes V(D)J recombination. These data show that modifications of H3K27me3 at the TCRβ locus likely contribute to an explanation of why the TCR repertoire is affected by gravity changes and imply, for the first time, EZH2 in the regulation of the TCRβ locus chromatin structure.

## 1. Introduction

During T lymphopoiesis, cells go through highly regulated differentiation steps that lead to the production of functional CD4+ and CD8+ T-cells. Four main differentiation stages can be identified using specific cell surface markers: the early double negative (DN) (CD4-CD8-), double positive (DP) (CD4+CD8+), single positive CD4 (SP4) (CD4+CD8-CD3+TCR+) and single positive CD8 (SP8) (CD4-CD8+CD3+TCR+) stages. The diversity of T-cell receptors (TCRs), composed of one heavy chain (TCRβ) and one light chain (TCRβ), is generated via the V(D)J recombination mechanism occurring at specific differentiation stages between the DN2 and DN3 stages for TCRβ at the DP stage for TCRβ. This V(D)J recombination process associates a variable (V) and a joining (J) gene segment to create an α chain gene and a variable (V), a diversity (D) and a joining (J) gene segment to create a β chain gene. V(D)J recombination relies on recombination signal sequences (RSS) located at the 3′ end of V, on both sides of D and at the 5′ end of the J segments, as well as on specific proteins (e.g., recombination-activating gene (RAG) proteins). Before V(D)J recombination, unrearranged V gene segments and J-C regions of the TCR loci are transcribed in a stage-specific manner [1,2,3,4]. Germline transcription of these loci, which is indicative of chromatin opening, is required for V(D)J recombination [3].

In addition to germline transcription, epigenetic and, more precisely, posttranslational histone modifications contribute to T lymphopoiesis [5,6] and V(D)J recombination [7]. Indeed, the histone methyltransferase PRC2 complex (Polycomb Repressive Complex 2), composed of EZH2 (Enhancer of Zeste Homolog), Suz12 (Suppressor of Zeste-12), EED (Embryonic Ectoderm Development) and RbAp46/48 (Retinoblastoma protein (Rb)-Associated protein (Ap)), is essential for T lymphopoiesis [8,9]. This complex, via EZH2 activity, adds between one to three methyl groups to the lysine 27 of histone H3 (H3K27me1–3). A specific knockdown of EZH2 in the T lineage induces a blockage of T-cell differentiation at the DN3 stage, corresponding to the β-selection checkpoint and the absence of mature T-cells [8,9]. At this stage, EZH2 methylates the Cdkn2a promoter to prevent p53 stabilization [10]. Different histone posttranslational modifications that participate in chromatin structure regulation and DNA boucle formation are also required to allow V(D)J recombination [11,12,13,14]. Indeed, Gopalakrishnan and colleagues observed correlations between the recombination efficiency, histone acetylation and H3K4 methylation [11]. However, no comprehensive data are available about the contribution of EZH2 and H3K27me3 to the regulation of TCRβ locus recombination under physiological or extreme conditions such as spaceflight.

During space missions, astronauts are exposed to different types of stresses, i.e., microgravity during flight, hypergravity at launch and landing, radiation, confinement, isolation, sleep deprivation and disrupted circadian rhythm, which can negatively affect their health [15,16]. Indeed, medical data collected from 46 astronauts who spent 6 months onboard the International Space Station (ISS) revealed that 46% of them presented immunological problems [17]. Benjamin et al. [18] evidenced a decrease in T-cell production in astronauts after a long-term space mission. Despite this important observation, studies about the effects of spaceflight on T-cell differentiation are rare [18,19,20,21,22,23]. Due to the cost and limited availability of spaceflight missions, various ground-based models have been developed to study the mechanisms underlying deleterious effects on the immune system. Among them, gravity change is widely studied, either by reducing gravity constraints by means of head down tilt bed rest for humans [24] and an anti-orthostatic suspension in rodents [25] or by increasing the gravity level through chronic centrifugation [26]. Even though they act mechanically in a opposite way, reducing both the gravity constraints and chronic centrifugation simulates many of the chronic stressors inherent to spaceflight experiments, i.e., exposure to a novel environment, changes in limbs loads, cephalic fluid distribution shifts and orthostatic intolerance [27]. Thus, using rotors to expose animals to different levels of hypergravity is an efficient means of understanding how altered gravity affects physiological functions. Furthermore, different studies demonstrated that micro- and hypergravity influence the shape of cells, initiate cytoskeleton reorganization and influence cell motility [28]. Concerning T-cell development, Woods et al. [19,20] showed that simulated microgravity impacts β-selection. Our team demonstrated that the repertoire of TCRβ chains is modified when animals develop in hypergravity (2× *g*) [21] or under environmental conditions mimicking the chronic socioenvironmental stressors encountered by astronauts [22], suggesting that TCRβ chain formation and/or selection processes are impacted by both types of stressors. Moreover, it was shown that, after 35 days spent in the ISS, mice presented a reduced thymic weight, and RNAseq analyses suggested lower proliferation capabilities of double-negative populations [23]. However, the molecular mechanisms underlying the TCRβ repertoire and T lymphopoiesis deregulations under spaceflight conditions are unknown. In this context, epigenetic deregulations in response to gravitational stress are interesting avenues to explore, as highlighted by recent studies that revealed increased H3K4me3 and H3K27me3 levels in microgravity-exposed human blood-derived stem cells [29] and lower H3K27me3 levels at specific promoters in human mesenchymal stem cells differentiated in vitro in microgravity [30]. These two studies suggest that H3K27me3 could be affected by gravitational stress in different cell types.

In this study, we wondered whether gravitational stress could impact V(D)J recombination by altering the H3K27me3 histone posttranslational modification and EZH2 methyltransferase activity. To this end, we exposed adult male mice to hypergravity (2× *g*) for 3 weeks to allow a comparison with the only previous study to have analyzed the effects of gravity change (2× *g* during 3 weeks) on the TCRβ chain repertoire and V(D)J recombination [21]. The 21-day duration was also defined by a similarity with the average duration (~6 months) of a mission in the ISS. Indeed, 6 months of human life would correspond to ~21 days in mice [31]. Our data revealed a global decrease in the thymic H3K27me3 level. However, local analysis revealed an increase in H3K27me3 on some specific regions of four Vβ segments in the murine double-negative SCIET27 thymic cell line exposed to 2× *g* gravity. Moreover, we demonstrated that EZH2 could contribute to V(D)J recombination regulation. Indeed, we showed that EZH2 inhibition in the SCIET27 cell line modified the expression of Vβ germline transcripts and that these changes correlated with a decrease in the H3K27 trimethylation on 5′ and 3′ ends of affected Vβ segments.

## 2. Results

### 2.1. Hypergravity Impacts T Lymphopoiesis in Adult Mice

**Figure 1:**
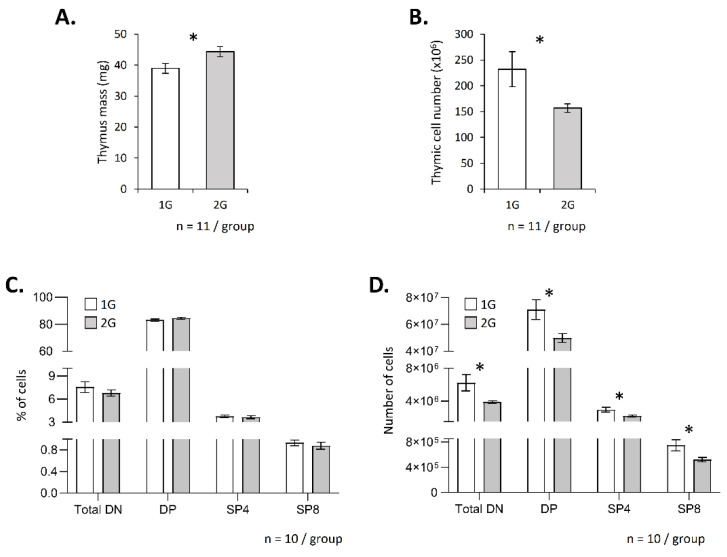
Hypergravity affects T lymphopoiesis. Thymus mass (**A**) and thymic cell number (**B**) in adult mice exposed to 2× *g* hypergravity for three weeks. (**C**,**D**) Analysis of T lymphopoiesis by flow cytometry. T-cell subpopulations were identified using CD4, CD8, CD3 and TCR staining. For each mouse, staining was done in duplicate, and mean was calculated. (**C**) Percentage of each subpopulation. (**D**) Absolute number of cells in each subpopulation. Data are the mean ± SEM of 11 (**A**,**B**) or 10 mice (**C**,**D**) per group. Mann–Whitney or *t*-tests were used to reveal statistically significant differences. * *p* < 0.05.

To evaluate the impact of hypergravity on T-cell development, we exposed adult male mice to 2× *g* for 3 weeks and then analyzed the mass and cellularity of their thymus. We observed that thymus mass slightly increased in hypergravity mice (Figure 1A), but its cellularity decreased (Figure 1B). Flow cytometry analyses did not reveal modification of the thymocyte subpopulation distribution; the percentages of the DN, DP, SP4 and SP8 were similar in 1G and in 2G mice (Figure 1C, Supplementary Figure S1). However, absolute cell numbers of the four subpopulations studied here decreased in the thymus of 2G mice (Figure 1D). These data suggest a potential global negative effect of hypergravity on T-cell development.

In search of an explanation to these decreases in the absolute cell number, we quantified serum corticosterone, a major stress hormone known to induce thymocyte apoptosis. No significant increase in circulating corticosterone was observed in 2G mice (Supplementary Figure S2). Most 2G mice had a corticosterone concentration similar to that observed in the 1G mice, though three 2G mice did deviate from the 1G mice. To determine the impact of the higher corticosterone concentration observed in these three animals, we compared the T-cell phenotypes of 2G mice with a high corticosterone concentration to the phenotypes observed in 2G mice with normal corticosterone levels. As shown in Supplementary Figure S3, we observed no significant difference between the two groups when analyzing cell percentages and only a slight increase in the SP8 when analyzing absolute cell numbers. Thus, changes in thymocyte subpopulations cannot be attributed to an increase in corticosterone. We also evaluated global apoptosis by studying the PARP1 cleavage. As shown in Supplementary Figure S4A,B, no increase in the PARP1 cleavage was observed in the 2G mice, suggesting no effect on thymocyte cell death. This observation was supported by measurement of the γH2A.X level, which is a marker of DNA double-strand breaks. As shown in Supplementary Figure S4C,D, no significant variation was detected. Finally, we wondered whether alterations in T lymphopoiesis could be due to changes in cell proliferation. To address this question, we performed an analysis of Cyclin D1 mRNA expression in thymocytes from eight mice exposed to either 1× *g* or 2× *g* gravity. As shown in Supplementary Figure S5, we did not detect any variation in its expression, suggesting an absence of cell cycle modification. Taken together, these results suggest that thymocyte development is negatively impacted by hypergravity and that this negative impact is independent of glucocorticoid-induced apoptosis.

### 2.2. Hypergravity Affects the TCRβ Repertoire of Adult Mice

**Figure 2:**
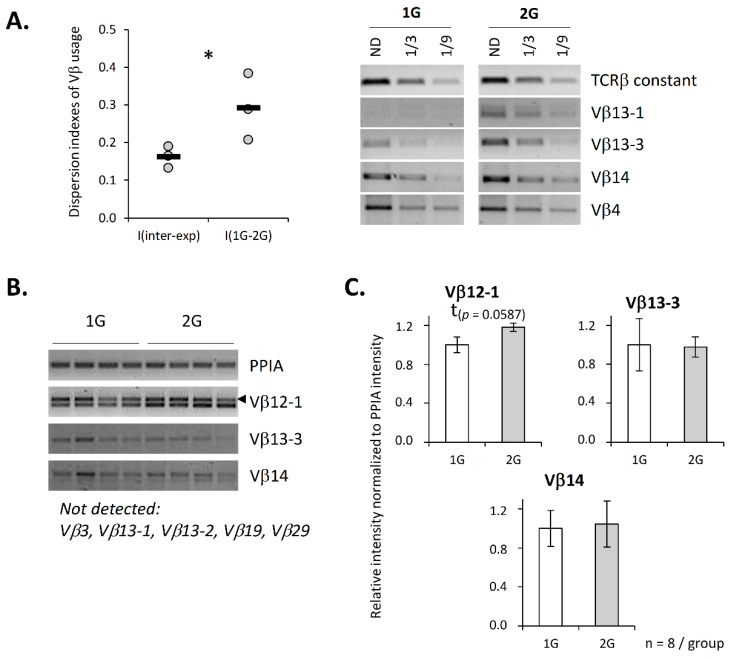
Hypergravity affects Vβ segments’ usage and germline transcripts. (**A**) Vβ segments’ expression was evaluated by semiquantitative PCR. Each experiment was performed using a pool of mRNA from 8 to 11 mice. The relative intensity for each of the 11 studied Vβ segments was normalized over TCRβ constant region amplification. The right panel presents the Vβ segments’ amplification results from one representative experiment out of three. Then, dispersion indexes were calculated to evaluate variation between experiments (I(inter-exp), used as reference) and to evaluate variation between 1G and 2G conditions in each experiment (I(1G–2G)). The graphic (left panel) presents dispersion indexes calculated for each experiment (dot) and the mean of three independent experiments (black line). ND = not diluted. (**B**,**C**) Expression of Vβ germline transcripts evaluated by semiquantitative PCR. (**B**) Example of amplification observed for four mice in each group. (**C**) Histograms presenting the relative intensity of Vβ germline transcripts amplification normalized to PPIA intensity. A total of 8 Vβ germline transcripts were tested, and only four were detected under our conditions. Data are mean ± SEM of eight mice per group. Mann–Whitney tests were used to reveal statistically significant differences. * *p* < 0.05, t indicates a tendency.

Since we previously showed that the TCRβ repertoire of pups developed under 2× *g* hypergravity conditions is altered [21], we checked that this repertoire is also affected when adult mice are exposed to this level of hypergravity. To this end, and to avoid performing a lengthy and thorough description of their repertoire by NGS sequencing, which is not the scope of this paper, we checked that the use of the 11 most common Vβ segments is affected in 2G mice by assessing, using semiquantitative PCR, the Vβ mRNA expression that was normalized to the total TCRβ mRNA. This normalization indicates an abundance of Vβ segments among TCRβ mRNAs. Since variation in the Vβ segment usage can be animal-dependent, we calculated dispersion indexes to assess repertoire variation, as described in Fonte et al. [22]. These indexes were used to perform two types of comparisons. First, we compared variations between the experiments (I(inter-exp)) to evaluate the normal variation of the Vβ segments independently of treatment. Second, we evaluated variations between the 1G and 2G conditions (I(1G–2G)) to determine whether hypergravity affects the TCRβ repertoire. Figure 2A shows that the indexes comparing the 1G and 2G repertoires, I(1G–2G), were significantly higher than the interexperiment indexes, I(inter-exp), which indicates that 2G exposure also modifies the TCRβ repertoire in adult mice.

Knowing that chromatin compaction contributes to the regulation of V(D)J recombination, we then asked whether chromatin compaction is changed under hypergravity exposure. To address this question, we quantified, by semi-quantitative PCR, some germline transcripts that are associated with chromatin opening. Three germline transcripts were detected among the eight that were tested. We evidenced a trend (*p* = 0.0587) toward an increase in Vβ12-1 germline transcripts in the thymus of the 2G mice (Figure 2B,C). This increase is in accordance with a modification of the chromatin structure and could suggest a modification of the repertoire due to chromatin opening at the level of specific Vβ gene segments.

### 2.3. Hypergravity Affects Tri-Methylation of Histone H3 Lysine 27 and EZH2 Level

**Figure 3:**
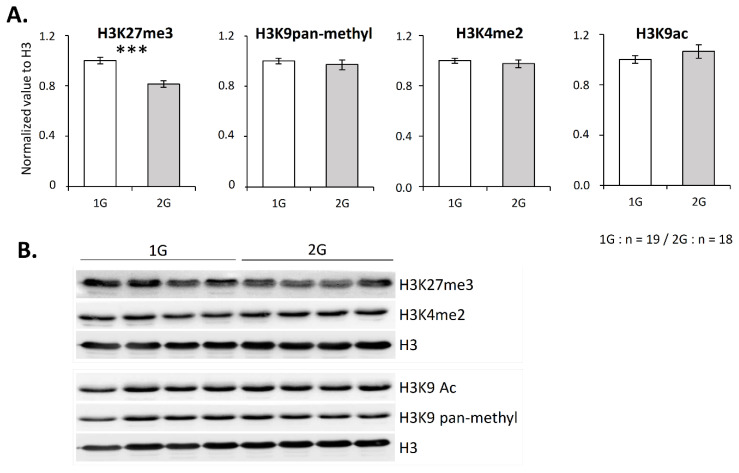
H3K27 trimethylation level is reduced in the thymus after 3 weeks spent at 2G. (**A**,**B**) H3 lysine 27 trimethylation, global H3 lysine 9 monomethylation, dimethylation or trimethylation (named panmethylation), H3 lysine 4 di-methylation and H3 lysine 9 acetylation evaluated by Western blotting. (**A**) H3-corresponding lysine methylation or acetylation relative expression normalized to H3. (**B**) Representative Western blots obtained with four mice in each group. Data are mean ± SEM of 19 or 18 mice per group. Mann–Whitney or *t*-tests were used to reveal statistically significant differences. *** *p* < 0.001.

As chromatin compaction is notably regulated by histone post-translational modifications, which contribute to T-cell development regulation [32] and V(D)J recombination [11,12,13,14], we wondered if hypergravity could impact some of these modifications. To address this question, global levels of four histone H3 modifications were evaluated by Western blotting. We targeted two methylations associated with chromatin closing (H3K27me3 and H3K9 pan-methylated) and two modifications associated with chromatin opening (H3K4me2 and H3K9ac). As shown in Figure 3, only H3K27me3 was significantly reduced (−20%) in the thymus of 2G mice.

**Figure 4:**
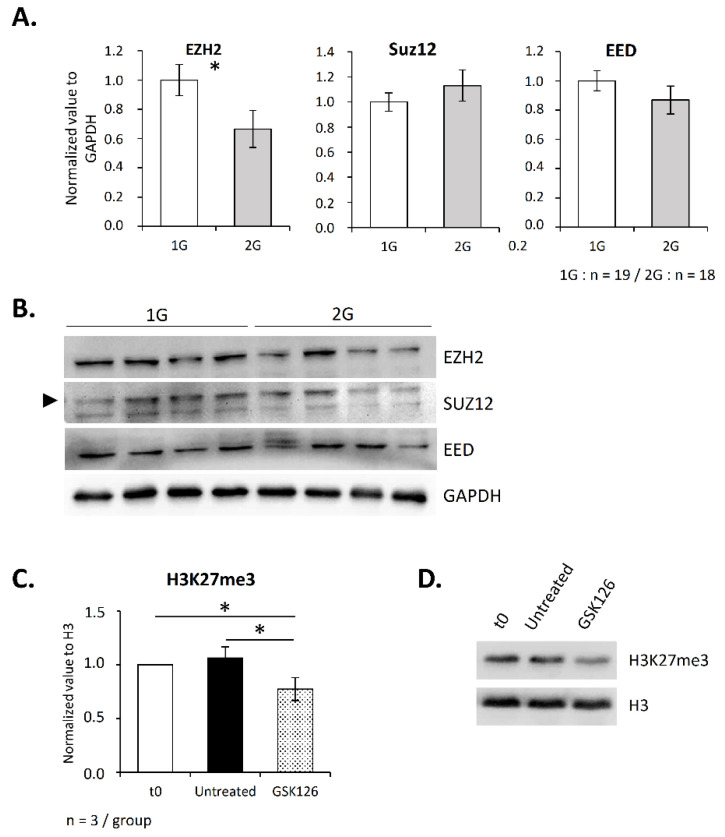
EZH2 is downregulated in the thymus after three weeks spent at 2× *g* and regulates H3K27 methylation in thymocytes. (**A**,**B**) Expression of PRC2 components (EZH2, Suz12 and EED) studied by Western blotting. (**A**) Relative expression of EZH2, Suz12 and EED normalized to GAPDH. (**B**) Representative Western blots obtained with four mice in each group. Arrow indicates Suz12 protein. (**C**,**D**) To evaluate the contribution of EZH2 activity to the H3K27me3 level in the thymus, thymocytes were exposed, or not, to the specific inhibitor GSK126 at a concentration of 2 µM for 10 h, and histones were extracted to perform Western blots. Here, ‘t0’ indicates H3K27me3 level in thymic cells before treatment. (**C**) Histogram presenting the relative expression of H3K27me3 normalized to H3. (**D**) Western blot representative of three independent experiments. Data are mean ± SD of three independent experiments. ANOVA was used to reveal statistically significant differences. * *p* < 0.05.

Knowing that the methyltransferase EZH2 is essential for T lymphopoiesis and that it is one of the two enzymes responsible for H3K27 methylation, we then asked whether its thymic expression is altered under hypergravity. As shown in Figure 4A,B, the EZH2 level was significantly decreased in the thymus of 2G mice. As EZH2 is part of the PRC2 complex, we also evaluated the level of its partners Suz12 and EED. Interestingly, their levels were not affected (Figure 4A,B). These observations suggest that the H3K27me3 decrease was a consequence of the EZH2 protein level decrease.

However, because few data exist concerning the contribution of EZH2 to H3K27methylation in the thymus, we wished to ensure that EZH2 is involved in this process. To this end, we treated thymocytes overnight with GSK126, which specifically inhibits EZH2 activity, and then assessed the level of H3K27me3 by Western blotting. As shown in Figure 4C,D, GSK126 induced a decrease in the H3K27me3 level (−20%). This result confirms that EZH2 participates in H3K27 methylation in the thymus, thus supporting the fact that 2G-induced alterations in H3K27me3 could be due to a modification in the EZH2 level.

Altogether, these results show that only some post-translational histone modifications are globally affected by hypergravity in the thymus. Furthermore, the decrease in H3K27me3 suggests a global alteration in the thymocytes’ chromatin structure in response to hypergravity, which could be explained by an alteration in the EZH2 level.

### 2.4. Hypergravity Modifies H3K27me3 Level along Vβ Segments in the DN2 SCIET27 Cell Line

**Figure 5:**
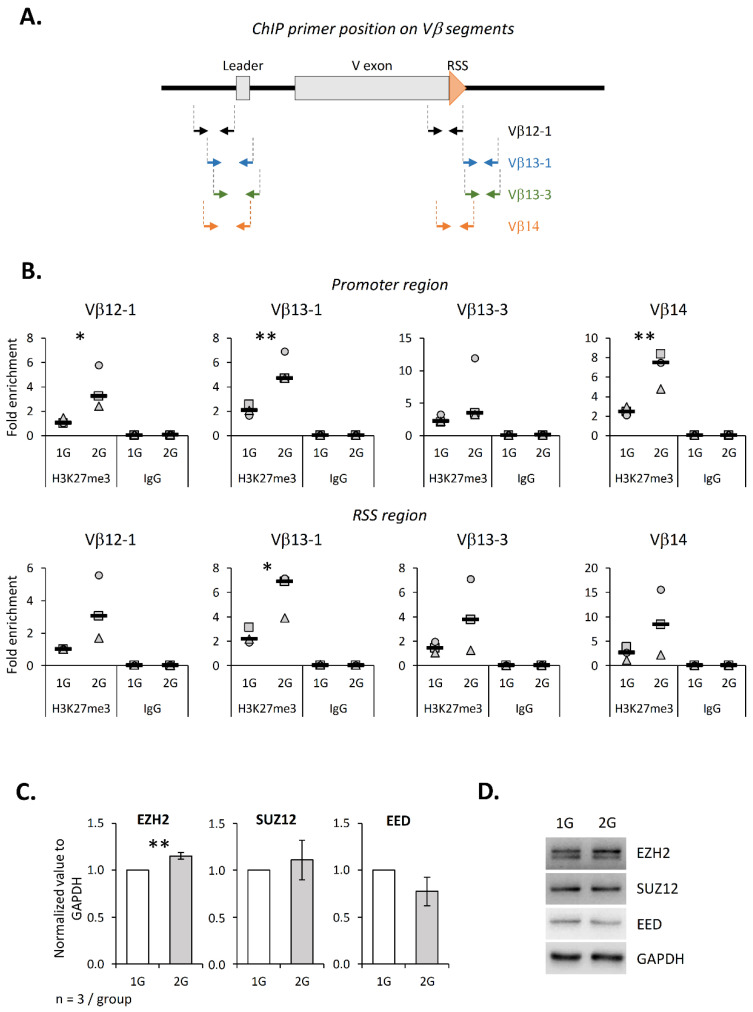
Hypergravity induces H3K27 tri-methylation increase at Vβ regions in the SCIET27 cell line. (**A**) Schematic representation of primer positions along one Vβ gene segment. Vβ12-1, Vβ13-1, Vβ13-3 and Vβ14 segments are composed of two exons (gray). Recombination signal sequences (RSS) are localized at the 3′ end of the V exon (orange triangle). Two pairs of primers were used for each Vβ segment; one localized on the promoter region and one localized near the RSS. (**B**) The presence of trimethylated H3K27 on the four Vβ segments was analyzed by ChIP. Graphics present the fold enrichment of H3K27me3 on the eight different chromatin regions. Symbols correspond to three independent experiments. Median is indicated as a black line. (**C**,**D**) Relative expression of PRC2 components (EZH2, Suz12 and EED) studied by Western blotting and normalized to GAPDH. (**C**) Levels of EZH2, Suz12 and EED normalized to GAPDH. (**D**) Western blots representative of three independent experiments. Data are mean ± SD of three independent experiments. *T*-tests or Mann–Whitney tests were used to reveal statistically significant differences. * *p* < 0.05, ** *p* < 0.01.

We then analyzed more precisely the consequence of hypergravity on the TCRβ locus. To this end, we exposed the murine DN2 SCIET27 cell line to 2× *g* during 18 h and performed ChIP experiments to evaluate the H3K27me3 level on the promoter and RSS regions of four Vβ segments (Vβ12-1, Vβ13-1, Vβ13-3 and Vβ14) (Figure 5A). This cell line was chosen because it is the last maturation stage before V(D)J recombination. Our results show that the H3K27me3 level was increased at the promoter of Vβ12-1, Vβ13-1 and Vβ14 and at the RSS of Vβ13-1 segments (Figure 5B), suggesting that hypergravity induced the local chromatin compaction. Since we observed a correlation between the EZH2 and H3K27me3 levels in the thymus from 2G mice (see above), we also evaluated the EZH2 level in SCIET27 exposed to 2× *g*. As shown in Figure 5C,D, EZH2 significantly increased in the 2× *g* condition, while no variations for Suz12 and EED were detected. However, in SCIET27, no global variation ifor H3K27 was detected (Supplementary Figure S6). Altogether, these results suggest that the methylation of H3K27 was locally deregulated in the SCIET27 cells exposed to 2× *g* hypergravity, notably at the promoter of the Vβ segments, which could be due to an increase in the EZH2 protein expression.

### 2.5. EZH2 Inhibition Alters Vβ Germline Expression via a Decrease in H3K27me3 in SCIET27 Cells

**Figure 6:**
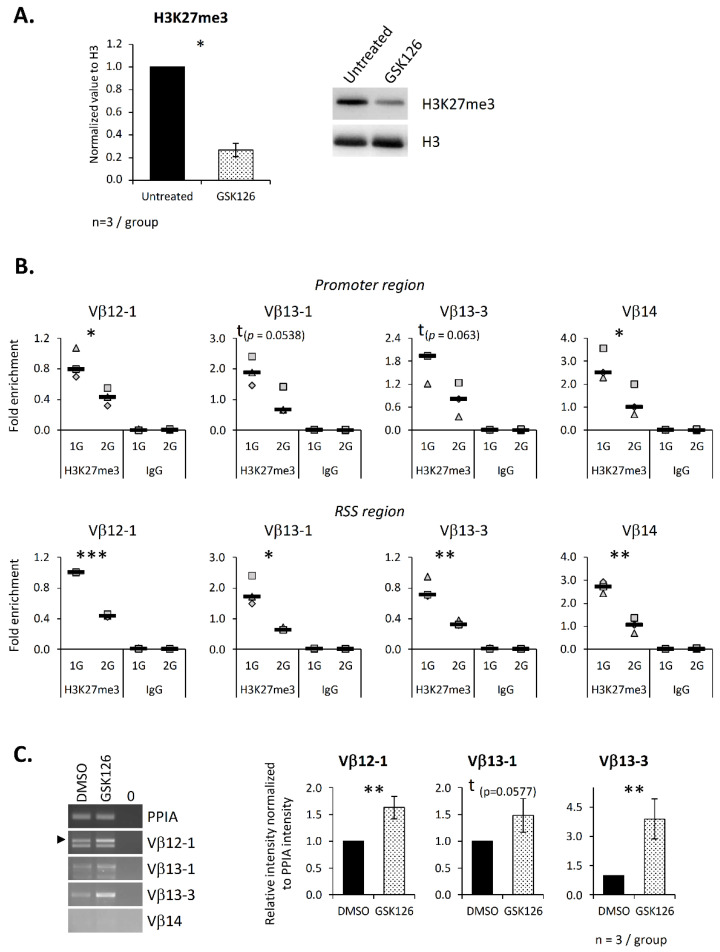
EZH2 inhibition alters germline transcript expression due to a decrease in H3K27me3 on the Vβ segment promoter and recombination signal sequence regions. The SCIET27 cell line was treated overnight with the specific inhibitor GSK126 at a concentration of 2 µM. Both the mRNA and histones were extracted, and ChIP was performed. (**A**) To evaluate the efficiency of GSK126 inhibition, the H3K27me3 level was quantified by Western blotting. The right panel shows one representative Western blot out of three independent experiments. The left panel presents the relative expression b of H3K27me3 normalized to histone H3 used as a loading control. (**B**) The presence of trimethylated H3K27 on the promoter and the RSS regions of four Vβ segments was analyzed by ChIP. Graphics present the enrichment of H3K27me3 on the eight different chromatin regions. Symbols correspond to three independent experiments. Median is indicated as a black line. (**C**) Expression of four Vβ germline transcripts evaluated by semiquantitative PCR. Left panel, representative experiment out of three independent experiments. Right panel, relative intensity of the Vβ germline transcripts’ amplification normalized to PPIA intensity. Data are mean ± SD of three independent experiments. Mann–Whitney tests (**A**,**C**) or *t*-tests (**B**) were used to reveal statistically significant differences. * *p* < 0.05, ** *p* < 0.01, *** *p* < 0.001, t indicates a tendency.

To ensure that EZH2 contributes to chromatin structure regulation at the TCRβ locus, the SCIET27 cell line was treated with the GSK126 inhibitor. This treatment induced an 80% decrease in the H3K27me3 level (Figure 6A), confirming that EZH2 is the main actor in H3K27 methylation in these cells. Next, ChIP experiments were performed, and the H3K27me3 level was evaluated at the same DNA regions as in Figure 5. Again, we detected the presence of H3K27me3 at all four studied Vβ segments. Comparison of untreated and treated cells showed a decrease in the H3K27 trimethylation on the RSS regions of the Vβ12-1, Vβ13-1 Vβ13-3 and Vβ14 segments, a significant decrease on the promoter regions of Vβ12-1 and Vβ14 and trends for a decrease on the promoter regions of the Vβ13-1 and Vβ13-3 segments (*p* = 0.0538 and 0.063, respectively) (Figure 6B). We then asked whether EZH2 contributes to the V(D)J recombination process. To this end, we analyzed the expression of the four Vβ germline transcripts studied in the ChIP experiments. Our results indicate that EZH2’s inhibition induced a significant increase in Vβ12-1 and Vβ13-3 germline transcripts and a trend (*p* = 0.0577) toward an increase in Vβ13-1 germline transcripts (Figure 6C). Note that Vβ14 germline transcripts could not be detected in this cell line.

Altogether, these results suggest that in some cases EZH2, via regulation of the H3K27me3 level on the Vβ segments, might contribute directly to germline transcript expression but that, in other cases (Vβ14), other actors are required. Indeed, we could not detect Vβ14 germline transcripts but could detect a decrease in H3K27me3 on its promoter and RSS regions. This observation could be due to the fact that a histone tail can carry different types of modifications acting together to generate histone code.

## 3. Discussion

Only a few studies have investigated the effects of spaceflight on lymphocyte development [18,19,20,21,22,23,33,34,35]. This study aimed to determine the impact of chronic gravitational change on murine T-cell development and to understand the molecular mechanisms responsible for TCRβ repertoire modification.

We found that 21 days spent at 2× *g* induced an increase in the murine thymus mass but decreased its cellularity. This increase in the thymus mass was also observed in male pups developed under hypergravity (2G) conditions [21]. Several studies have reported the impact of spaceflight on mouse and rat thymus masses, but the conclusions have been inconsistent. The mass of this organ has been reported to decrease [23,36], increase [37] or remain constant [38,39]. Such observations might be due to variable experimental conditions encountered during space missions. We noted that this increase in thymus mass was associated with reductions in lymphocyte absolute numbers at all stages of T-cell development. This observation contradicts the results reported by Tateishi et al. [40], who did observe a decrease in the murine C57BL/6 thymus mass and cellularity. This discrepancy could be explained by the difference in hypergravity exposure duration; our mice were exposed for 3 weeks versus 2 weeks for Tateishi and colleagues [40]. Horie and collaborators [23] also noted a decrease in thymocytes and attributed this decrease to an increase in corticoid production during spaceflight, which is able to stimulate thymocyte apoptosis. In our case, we did not detect significant increases in serum corticosterone, as previously noted by Guéguinou et al. [41], nor in apoptosis in the thymus of hypergravity mice, suggesting that the negative impact of hypergravity on thymocyte development could involve glucocorticoid-independent pathway(s). However, we cannot exclude this possibility, as we only quantified the corticosterone in sera, and not the glucocorticoids produced inside the thymus, and the detection of apoptosis is very difficult in the thymus due to the continuous clearance of apoptotic cells by the macrophages [42,43,44]. Another hypothesis to explain the decrease in cellularity is cell cycle arrest. Indeed, spaceflight seems to impact the regulation of cell cycle factors [23] but the quantification of Cyclin D1 mRNAs does not support this hypothesis. However, again, we cannot exclude this possibility because our cell cycle study is limited and our results suggest a modification of EZH2 activity, which is known to be involved in cell cycle regulation, autophagy, apoptosis and cellular senescence [45,46,47]. Taken together, these data indicate that different phenomena are likely responsible for the thymocyte number decreases under hypergravity conditions.

A likely explanation for a global lymphocyte decrease is a modification of thymic microenvironment, which is essential for T-cell development. Different cell types contribute to this microenvironment, including thymic epithelial cells (TECs) whose number and functions have been shown to be reduced in hypergravity mice [40]. Additionally, TEC development and function are partly regulated by the PRC2 complex [48]. PRC2 inactivation in TECs has been shown to induce a modification of the TCRβ chain diversity in the SP4 and SP8 cells [48]. In accordance with these observations, we observed a global decrease in H3K27me3 in the thymus of 2G mice, suggesting an alteration of the PRC2 activity. Consequently, PRC2 could perhaps also be altered in TECs, thereby modifying their development and function and, consequently, T lymphopoiesis.

At the molecular level, epigenetics contributes to V(D)J recombination regulation [11,12,13,14]. Our previous study suggested that hypergravity modifies the TCRβ repertoire by affecting the chromatin structure at the level of the TCRβ locus [21]. Our germline transcripts analyses performed in the thymus of the 2G mice support this hypothesis, as they suggest chromatin remodeling and, more precisely, chromatin opening. Interestingly, microgravity-exposed human blood-derived stem cells were shown to present an increase in H3K27me3 [29], while a decrease in H3K27me3 at specific promoters was observed in human mesenchymal stem cells differentiated in vitro in microgravity [30]. These data show that the level of H3K27me3 is impacted by gravitational stress in various cell types and that this level could vary locally or globally. Consequently, we studied the impact of hypergravity on the promoter and RSS regions of four Vβ segments in the SCIET27 cell line. Interestingly, we observed a significant increase in H3K27me3 at three Vβ promoter regions in response to hypergravity exposure, while only one RSS region presents a significant increase in H3K27me3, which shows that modifications occur preferentially at the promoter region. This result is apparently in contradiction with our global analysis on thymocytes. However, it is important to note that a global decrease noted at the level of a tissue cannot predict a decrease at specific DNA regions in the cell type composing this tissue. We performed our ChIP experiments on the SCIET27 cell line and not on primary thymocytes because DN2 cells (the population equivalent to the SCIET27 cell line) is a rare population. As a result, our overall analysis is not predictive of what is happening in this subpopulation. Consequently, in the future, it would be very interesting to study the H3K27me3 distribution along the genome in different thymic subpopulations to determine whether the H3K27me3 landscape is altered in response to a gravity change, with a focus on the DN2 subpopulation to evaluate the status of the TCRβ locus before V(D)J recombination. However, given that chromatin is regulated by a large combination of histone posttranslational modifications, it will also be necessary to enlarge epigenetic studies to include more modifications, such as H3K4me3, which is associated with chromatin opening and transcription, in contrast to H3K27me3.

Different enzymatic activities are implicated in the regulation of H3K27 methylation. PRC2, via EZH2 or its homolog EZH1, adds methyl groups, while KDM6a and KDM6b demethylate this lysine [49]. In this study, we concentrated our attention on EZH2, as it is known to be essential for T-cell development [8,9]. Furthermore, EZH2 is the main effector of H3K27 methylation in DN subpopulations [8,10], as confirmed by our treatment of the SCIET27 DN2 murine cell line with the GSK126 inhibitor. Our results suggest a direct implication of EZH2 in TCRβ’s chromatin structure regulation. Indeed, we observed specific decreases in H3K27 trimethylation at 5′ and 3′ ends of four Vβ segments in response to the GSK126 treatment. These results, combined with the germline transcripts’ data, suggest an implication of EZH2 in the regulation of V(D)J recombination while the non-exhaustive study of Su et al. evidenced no implication in V(D)J recombination [8].

Finally, we showed that in the SCIET27 cell line, hypergravity induced a global increase in EZH2 protein level, which was not correlated with an increase in H3K27me3. This absence of correlation could be due to a modification of the balance between the methylase (EZH2/EZH1) and demethylase (KDM6a/b) activities that coexist within cells. To our knowledge, no data currently exist concerning KDM6a/b’s function in the thymus. Thus, it would be very interesting to test this hypothesis in the future. Another hypothesis is a modification of EZH2’s function. Indeed, EZH2 can methylate H3K27, but it can also act on nonhistone proteins, such as talin [50] or promyelocytic leukemia zinc finger protein (PLZF) [51]. Moreover, it has recently been shown that EZH2 is involved in the regulation of centrosome polarization in SCIET27 cells [52]. As cytoskeletons are impacted by gravitational force alteration [53,54,55,56], EZH2 could perhaps interact differently with its targets to contribute to cell adaptation, which could explain this absence of correlation. Consequently, in the future it will be interesting to study the impact of gravitational forces on the different functions of EZH2 in the various subpopulations of thymocytes.

Note that this study is limited by the fact that we could not quantify the glucocorticoids produced within the thymus; the lack of quantification of hormones, such as ACTH, CRF and catecholamines; the difficulty in detecting apoptosis in the thymus; a limited study of cell cycle; and the fact that the balance between methylase (EZH2/EZH1) and demethylase (KDM6a/b) activities was not studied.

In conclusion, our results show for the first time that the level of a specific histone modification, H3K27me3, is decreased in the murine thymocytes following hypergravity exposure and that these alterations will probably induce epigenome modifications that contribute to a decrease in the absolute T-cell number. In the future, it will be interesting to study more extensively the epigenomes of various thymocyte subpopulations to improve our understanding of alterations in T lymphopoiesis in response to gravity changes, especially under simulated or real microgravity conditions, and pay more attention on the TCR loci structure to understand how its modification will impact the TCR repertoire’s creation.

## 4. Materials and Methods

### 4.1. Animals

Experimental procedures were carried out in conformity with the French National Legislation and the Council Directive of the European Communities on the Protection of Animals Used for Experimental and Other Scientific Purposes (2010/63/UE). Experiments were approved by the French Ministry of Research (authorization 04827), and authors complied with the ARRIVE guidelines. The mice used in this study were 7-week-old male C57BL6/J mice purchased from Charles River (L’arbresles, France). For acclimation to animal house conditions, mice were housed for a week in standard cages (four mice per cage, 36 cm × 20 cm × 14 cm) in a quiet room under constant conditions (22 °C, 50% relative humidity, 12-h light/dark cycles) with free access to standard food and water. Animals were anesthetized using 5% isoflurane and then put to death by cervical dislocation.

### 4.2. Mice Hypergravity Exposure

Standard cages (36 cm × 20 cm × 14 cm) containing four mice were placed in a large radius centrifuge (radius of 1.80 m) [57] with a rotational speed of 32 rpm, producing a gravity vector of 2× *g* (Supplementary Figures S7 and S8). Mice were supplied with enough food and water for 3 weeks so that the centrifuge was operating continuously. Mice were left undisturbed during the 3 weeks of centrifugation. An infrared video allowed remote day and night control of the mice in gondolas. All environmental variables, except the gravity level, were the same as in standard housing. The cages containing control mice were placed in opaque gondolas similar to and in the same room as the centrifuged mice but in a static position. At the end of the 21 days of centrifugation, control and 2G mice were immediately anesthetized using 4% isoflurane and put to death for biological sample collection.

### 4.3. Antibodies, Reagents and Cell Line

Anti-CD4 PE (RM4-5), anti-CD8a PECy7 (53-6.7), anti-TCRβ FITC (H57-597), anti-CD3 APC (17A2) and the respective isotype controls were purchased from Biolegend (Ozyme, Saint-Quentin-en-Yvelines, France). Anti-GAPDH (G9545) and anti-EZH2 (07-689) used for Western blot, and anti-EED (AA19), anti-H3K27me3 (07-449), anti-H3K4me2 (07-030) and anti-H3K9ac (07-352) antibodies were purchased from Merck Millipore (St.-Quentin-en-Yvelines, France). Anti-Suz12 (D39F6) and anti-H3K9-panmethyl (4069) antibodies were purchased from Cell Signaling (Danvers, MA, USA). The anti-γH2A.X (39118) and the anti-H3 C-terminal (39164) antibodies were obtained from Active Motif (La Hulpe, Belgium). Anti-PARP1 antibody (GTX100573) came from Gentex (Zeeland, MI, USA). The EZH2 inhibitor GSK126 was purchased from Interchim (Montluçon, France) (Table S1).

The SCIET27 cell line was kindly provided by I. Screpanti (Laboratory of Molecular Pathology, Sapienza University of Rome, Rome, Italy) and I. Aifantis (New York University, School of Medicine, New York, NY, USA) (Table S1).

### 4.4. Flow Cytometry

Thymuses were dissociated in PBS−2% FCS, and cells were counted using a Scepter Cell Counter (Merck Millipore, St.-Quentin-en-Yvelines, France). To identify DN, DP, SP4 and SP8 subpopulations, cells were stained with anti-CD4 PE, anti-CD8a PE-Cy7, anti-TCRβ FITC and anti-CD3 APC (Figure S1A,B). This staining was also used to evaluate putative contamination in the DN subpopulation (Figure S1C). An amount of 5 × 10^5^ fresh cells were stained for 30 min in PBS−2% FCS at 4 °C and then washed in PBS−2% FCS. Data acquisition was performed using a Gallios Beckman Coulter flow cytometer, and data analysis was performed using FlowJo software (TreeStar, Inc., Ashland, OR, USA).

### 4.5. Serum Corticosterone

Serum corticosterone concentration was measured in duplicate using a commercial ELISA kit (Corticosterone Enzyme Immunoassay Kit, Arbor Assays, Euromedex, France), according to manufacturer’s instructions, and serum samples were prepared from blood collected between 8 and 10 a.m.

### 4.6. Cell Culture, EZH2 Inhibition and Hypergravity Exposure

The SCIET27 cell line was cultured in IMDM (Gibco, Thermo Fisher, Waltham, MA, USA) supplemented with 0.1 mM penicillin, 0.1 mM streptomycin (Sigma-Aldrich, Saint-Quentin Fallavier, France) and 10% FCS (Gibco, Thermo Fisher) according to Aifantis et al. 2001 [58].

For EZH2 inhibition, SCIET27 cells were plated at 1.5 × 10^6^ cells/mL in IMDM medium supplemented with 10% FCS, 0.1 mM penicillin and 0.1 mM streptomycin in the absence or presence of 2 µM GSK126 inhibitor and incubated overnight at 37 °C and 5% CO_2_.

For hypergravity exposure, SCIET27 cells were plated at 1.5 × 10^6^ cells/mL in IMDM medium supplemented with 10% FCS, 0.1 mM penicillin and 0.1 mM streptomycin in the Petaka^®^G3 culture device (Celartia, Columbus, OH, USA). Then, the Petaka^®^G3 devices were placed overnight in a centrifuge (radius of 10.5 cm) with a rotational speed of 132 rpm producing a gravity vector of 2× *g*, which was placed in an incubator set at 37 °C (Supplementary Figure S9). Control Petaka^®^G3 devices were placed in the same incubator but not subjected to hypergravity exposure.

Histones and total RNA were extracted from SCIET27 cells subjected to EZH2 inhibition or hypergravity exposure as described below, and ChIP was performed.

### 4.7. Total RNA Extraction and Reverse Transcription

RNA was extracted from the thymus of hypergravity and control mice using the RNeasy kit (Qiagen, Courtaboeuf, France) and from 10 × 10^6^ dissociated thymocytes using the All prep DNA/RNA/Protein Mini Kit (Qiagen, Courtaboeuf, France). In both cases, RNA was reverse transcribed using random primers, RNaseOut and MML-V reverse transcriptase (Invitrogen, Cergy Pontoise, France), following the manufacturer’s instructions.

### 4.8. Quantitative PCR

The qPCRs were performed in triplicate using Takyon No ROX SYBR MasterMix blue dTTP (Eurogentec, Liège, Belgium) and a Mastercycler Realplex2 Real-Time PCR System (Eppendorf, Hamburg, Germany). The cycling program was: 5 min at 95 °C followed by 40 cycles of 15 s at 95 °C and 45 s at the annealing temperature indicated in Table 1. Relative expression of Cyclin D1 transcripts was standardized using 3 housekeeping transcripts (EIF3F, RPL13A and PPIA) using a method previously described [22]. Primers used to amplify transcripts were designed in different exons to avoid the amplification of potential genomic DNA traces. Primer specificity was checked using a Basic Local Alignment Search Tool (BLAST) search through the US National Center for Biotechnology Information (Bethesda, MD, USA).

### 4.9. Germline Transcripts Amplification

Semiquantitative PCR amplifications were performed because some Vβ segments have highly homologous DNA sequences that prevent the design of specific primers that generate 150–200 bp amplicons, a prerequisite to perform real-time PCR. To ensure the specificity of our amplifications, we had to design primers in leader and V-encoding exons, thereby generating amplification products >200 bp. These polymerase chain reactions (PCRs) were done using 0.625 U of Taq Polymerase (Thermo Fisher) and 0.7 µM of each primer. The PCR program was 5 min at 95 °C, followed by cycles of 45 s at 95 °C, 45 s at the annealing temperature indicated in Table 2, 1 min at 72 °C and a final elongation at 72 °C for 10 min. The number of PCR cycles was chosen to be in the linear range of exponential amplifications. The PCR products were run on 1.5% agarose gels and visualized using the Fusion FX7 camera (Vilbert-Lourmat, Marne-la-Vallée, France). Quantifications of the amplification products were performed using ImageJ software (NIH) and normalized to PPIA amplification (housekeeping gene).

### 4.10. RACE-PCR and Vβ Segment Usage

TCRβ mRNAs were amplified by 5′-RACE PCR using the SMARTer™ RACE cDNA amplification kit (Takara Bio, Inc., Mountain View, CA, USA). Briefly, for each experiment or treatment, total RNA from the thymus or thymocytes of 8 or 12 mice (depending on the experiment) was mixed in equimolar quantities, and 200 ng of this mix were reverse transcribed according to the manufacturer’s instructions. Then, TCRβ mRNAs were preamplified via two successive PCRs using Advantage 2 Taq DNA polymerase (Takara Bio, Inc., Mountain View, CA, USA). The first PCR was performed using the GSP1 primer (Table 3) annealing to the TCRβ constant region and the UPM primer provided in the kit. The second reaction was a nested PCR performed using NGSP (specific to the TCRβ constant region but annealing upstream of GSP1) and NUP primers. Specific PCR products (approximately 650 bp) were purified using the NucleoSpin gel and PCR clean up kit (Macherey Nagel, Hoerdt, France), diluted and used as templates to perform Vβ-specific semiquantitative PCRs as explained above. These reactions were performed using Vβ-specific primers and the NGSP primer (Table 3 and Table 4). PCR products were run on a 1.5% agarose gel and visualized using an FX7 camera (Vilbert-Lourmat). Quantification of the amplification products was performed using ImageJ software (NIH) and normalized over TCRβ constant region amplification obtained using the TCRβ-For and NGSP primers. Then, each normalized signal was expressed as a percentage of the sum of the normalized signals obtained for the 11 studied Vβ segments. Then, these percentages were used to calculate dispersion indexes according to Fonte et al. [22]. These indexes were used to perform two types of comparisons. First, we compared variations between experiments (I(inter-exp)) to evaluate the normal variation in Vβ segment usage independently of the treatment and used this index as a reference. Second, we evaluated variations between 1G and 2G conditions (I(1G–2G)).

### 4.11. Total Protein Extraction

Proteins were extracted from thymus lysed in total buffer (10 mM HEPES pH 7.9, 0.4 mM NaCl, 1.5 mM MgCl_2_, 0.1 mM EGTA, 5% glycerol, 0.5% NP40) supplemented with Halt Protease and Phosphatase Inhibitor Cocktail following the manufacturer’s instructions (Thermo Fisher) at a ratio of 10 mg of tissue for 100 µL of total buffer. This lysis was performed on ice for 30 min. Then, tubes were centrifuged at 13,500× *g* for 15 min at 4 °C, and supernatants were collected. Proteins were extracted from dissociated thymocytes using the All prep DNA/RNA/Protein Mini Kit (Qiagen) following the manufacturer’s instructions.

### 4.12. Histone Extraction

Histones were extracted from thymuses homogenized in TEB buffer (0.2% Triton X-100 (*v*/*v*), 0.02% NaN_3_ (*w*/*v*), 5 mM sodium butyrate, 1× PBS) supplemented with Halt Protease and Phosphatase Inhibitor Cocktail (Thermo Fisher) at a ratio of 15 mg of tissue for 500 µL of TEB buffer for 10 min on ice with regular mixing. Histones were extracted from 5 × 10^7^ dissociated thymocytes by mixing them with 750 µL of TEB buffer and incubating the obtained solution on ice for 10 min. Then, in both cases, samples were centrifuged at 800× *g* for 10 min at 4 °C. Pellets were washed with TEB buffer (100 µL for the tissue, 150 µL for thymocytes) and incubated overnight with 0.2 M HCl at 4 °C (60 µL for the tissue, 30 µL for thymocytes). A final centrifugation at 6000× *g* for 10 min at 4 °C was performed, and the supernatants containing histones were recovered.

### 4.13. Western Blot

Total proteins or histone extracts were quantified using the Bradford method. To start, 10 to 30 μg of total proteins or 10 μg of histones were heated at 95 °C for 5 min, run on 10% SDS-polyacrylamide gels for proteins or 16% SDS-polyacrylamide gels for histones and transferred to PVDF (polyvinylidene difluoride) membranes (Amersham, Buckinghamshire, UK). Membranes were incubated with primary antibodies overnight at 4 °C and then with corresponding HRP-coupled secondary antibodies for 1 h at room temperature. Blots were stripped and probed again as necessary. An anti-GAPDH antibody or an antibody directed against the C-terminal tail of histone H3 was used as a loading control for total proteins or histones, respectively. Pierce ECL Western blotting substrate (Thermo Fisher) was used for immunodetection, and signals were visualized by chemiluminescence using a Fusion FX7 camera (Vilbert-Lourmat). Signal intensity was quantified using ImageJ software (NIH) and normalized over the corresponding loading control.

### 4.14. Chromatin Immunoprecipitation

ChIP was performed as described in Ghislin et al., 2012 [59] with some modifications. Cells were cross-linked by adding 10× fixation buffer (11% formaldehyde, 50 mM HEPES pH 8, 100 mM NaCl, 1 mM EDTA) at room temperature for 10 min. The cross-linking reaction was stopped by the addition of glycine to reach a final 1× concentration and incubation for 5 min at room temperature. After centrifugation (300× *g*, 5 min, 4 °C), the cells were washed in 1× PBS. Then, cell prelysis was performed as previously described [59]. Cells were lysed by incubation for 5 min on ice at 1.2 × 10^6^ cell/mL in lysis buffer (35 mM Tris-HCl pH 8.1, 5.8 mM EDTA, 75 mM NaCl, 0.3% Triton X-100, 0.5% SDS, 5 mM sodium butyrate) supplemented with Protease Inhibitor Cocktail (Sigma Aldrich, MO, USA). Cell lysates were diluted five times in sonication buffer (23 mM Tris HCl pH 8.1, 2.44 mM EDTA, 135 mM NaCl, 0.54% Triton X-100, 0.132% SDS, 5 mM sodium butyrate) supplemented with protease inhibitors; 300 µL of cell lysate were sonicated for 3 cycles of 30 s using the ultralow program of the Bioruptor Pico sonication device (Diagenode, Liège, Belgium) and then centrifuged for 10 min at 4 °C at maximum speed. Supernatant containing chromatin was collected. For immunoprecipitation, 50 µL of Dynabeads protein A (Invitrogen, Cergy Pontoise, France) were washed 3 times with IP Buffer (17.5 mM Tris-HCl pH 8.1, 1.3 mM EDTA, 162 mM NaCl, 1% Triton X-100, 0.02% SDS) and blocked with IP Buffer containing 5% BSA and 0.2 µg/µL of salmon sperm (Sigma Aldrich, MO, USA) for 2 or 12 h at 4 °C. Then, 250 µL of chromatin were diluted with 250 µL of IP buffer supplemented with 2.5% BSA and protease inhibitors. Chromatin was precleared by adding 20 µL of blocked Dynabeads and incubating for 1 h at 4 °C. Collected chromatin was then incubated overnight at 4 °C with 1.5 µg of antibodies (anti-H3K27me3 (C15410069, Diagenode) or isotype control (IgG control, C15410206, Diagenode)). Before adding antibodies, 2.5 µL of chromatin (input control) was collected and stored at 4 °C. Finally, immunoprecipitation was performed. A total of 30 µL of blocked Dynabeads were added to chromatin and incubated for 20 min at room temperature. Dynabead complexes were washed twice with low salt wash buffer (0.1% SDS (*v*/*v*), 1% Triton X-100 (*v*/*v*), 2 mM EDTA, 20 mM Tris-HCl pH 8.1), twice with high salt wash buffer (0.1% SDS (*v*/*v*), 1% Triton X-100 (*v*/*v*), 2 mM EDTA, 20 mM Tris-HCl pH 8.1, 500 mM NaCl), twice with LiCl wash buffer (250 mM LiCl, 1% NP-40 (*v*/*v*), 1% sodium deoxycholate (*v*/*v*), 1 mM EDTA, 10 mM Tris pH 8.1) and twice with TE buffer (10 mM Tris-HCl pH 8.1, 1 mM EDTA). Crosslinking was reversed by incubating Dynabeads complexes in elution buffer (10 mM Tris-HCl pH 8.1, 1 mM EDTA, 1% SDS (*v*/*v*)) overnight at 65 °C, followed by incubation at 37 °C for 1 h with proteinase K 0.5 mg/mL. DNA fragments were purified using NTB Buffer and the NucleoSpin Gel and PCR Clean-up kit (Macherey-Nagel, Hoerdt, France) following the manufacturer’s instructions. Quantitative PCRs (qPCR) were performed in triplicate using Takyon No ROX SYBR MasterMix blue dTTP (Eurogentec, Liège, Belgium) and a Mastercycler Realplex2 Real-Time PCR System (Eppendorf, Hamburg, Germany). The cycling program was 5 min at 95 °C followed by 40 cycles of 15 s at 95 °C and 45 s at the annealing temperature indicated in Table 5. Primers (Eurogentec, Liège, Belgium) were designed to amplify the promoter or the 3′ end of Vβ segments containing the recombination signal sequences. Primer specificity was checked using a Basic Local Alignment Search Tool (BLAST) search through the US National Center for Biotechnology Information (Bethesda, MD, USA).

### 4.15. Statistics

Statistical analyses were performed using StatView software (SAS Institute, Cary, NC, USA). For two-group comparisons, the homogeneity of variance was assessed with Fisher’s test and the normality of distribution was assessed with the Kolmogorov–Smirnov test. When both criteria were validated, *t*-tests were performed. When the variance and/or the distribution was not confirmed, Mann–Whitney nonparametric tests were performed. For more than two group comparisons, the homogeneity of variance and normality of distribution were checked using the Levene and Shapiro–Wilk tests, respectively. If these two sets of test criteria were met, ANOVA tests followed by post hoc Tukey–Kramer tests were performed for two by two comparisons. In the other cases, Kruskal–Wallis tests followed by post hoc Dunn tests were performed. Here, *p* values <0.05 indicate significance. All in vivo results are shown as the mean ± standard error of the mean (SEM). All in vitro results are shown as the mean ± standard deviation (SD).

## Figures and Tables

**Table 1:** Primers used to perform RT-qPCR.

Target	Sequences	Length (bp)	Annealing Temperature (°C)
PPIA	F 5′-GTCTCCTTCGAGCTGTTTGC-3′ R 5′-GCGTGTAAAGTCACCACCCT-3′	150	58
EIF3F	F 5′-CATCAAGGCCTATGTCAGCA-3′ R 5′-GTGGTGGGACTGTGTGTCTGG-3′	117	61
RPL13A	F 5′-GGAAGCGGATGAATACCAAC-3′ R 5′-CTTGTCATAGGGTGGAGGGA-3′	167	61
Cyclin D1	F 5′-TGACTGCCGAGAAGTTGTGC-3′ R 5′-CATCGAACACTTCCTCTCCA-3′	144	62

**Table 2:** Primers used to quantify TCRβ germline transcripts.

Target	Sequences	Length (bp)	Annealing Temperature (°C)
Vβ3	F 5′-ATGGATATCTGGCTTCTAGGT-3′ R 5′-GTTTGTGTACAGGAAGACGGT-3′	470	54
Vβ12-1	F 5′-CATCCTGAGAAGAAGCATGTC-3′ R 5′-TTACAGAAAGCCAGTAGCTTTG-3′	550	55
Vβ13-1	F 5′-CACTAAGTCACTGAAAGCCC-3′ R 5′-CCACACATCACTGTGCATC-3′	524	54
Vβ13-2	F 5′-TCCAGGCTCTTCTTCGTG-3′ R 5′-CCCCACATCACTGTGCATCA-3′	446	56
Vβ13-3	F 5′-AGCCCCAGTTCTAATTTACC-3′ R 5′-GAAGGAAGCCACACATCAC-3′	523	54
Vβ14	F 5′-GGCAGTGTTCTGTCTCCTTG-3′ R 5′-GAAAACCATCAGCTTTGTGC-3′	477	55
Vβ19	F 5′-AACAAGTGGGTTTTCTGCTG-3′ R 5′-ATGGAGAGGGGGTAGCTGT-3′	488	59
Vβ29	F 5′-GTTAGGCTCATCTCTGCTGTG-3′ R 5′-GAAAGGATGTGGCTGTGTAGA-3′	500	54
PPIA-2	F 5′-CACCGTGTTCTTCGACATCA-3′ R 5′-TTCTGTGAAAGGAGGAACCC-3′	155	62

**Table 3:** Primers used to perform RACE-PCR.

Target	Sequences	Annealing Temperature (°C)
GSP1	5′-CCCACTGTGGACCTCCTTGCCATTCACC-3′	68
NGSP	5′-CACGTGGTCAGGGAAGAAGCCCCTGGCC-3′	68
NUP	5′-AAGCAGTGGTATCAACGCAGAGT-3′	68

**Table 4:** Primers used to study Vβ usage in the TCRβ repertoire.

Target	Sequences	Length (bp)	Annealing Temperature (°C)
Vβ3	5′-ATGGATATCTGGCTTCTAGGT-3′	600 to 650 bp	60
Vβ4	5′-CTGTAGGCTCCTAAGCTGTG-3′		62
Vβ5	5′-GCTTCTCCTCTATGTTTCCCT-3′		60
Vβ13-1	5′-GGCTCTTTCTGGTCTTGAGC-3′		57
Vβ13-3	5′-CCAGACTCTTCTTTGTGGTTTT-3′		62
Vβ14	5′-GGCAGTGTTCTGTCTCCTTG-3′		54
Vβ17	5′-TGATCTTCTGTCTTCTTGCAG-3′		62
Vβ19	5′-AACAAGTGGGTTTTCTGCTG-3′		58
Vβ26	5′-GCTACAAGGCTCCTCTGTTA-3′		62
Vβ29	5′-GTTAGGCTCATCTCTGCTGTG-3′		60
Vβ31	5′-GGTGTTAGTGCTCAGACTATC-3′		54
TCRβ-For	5′-GATCTGAGAAATGTGACTCCAC-3′	120	60

**Table 5:** Primers used for ChIP experiments.

Target		Sequences	Length (bp)	Annealing Temperature (°C)
Promoter	Vβ12-1	F 5′-CACAGAAGGGCATAGCCAAC-3′ R 5′-TGCTGGTCCTCTTGGTGAGA-3′	179	61
	Vβ13-1	F 5′-CACTAAGTCACTGAAAGCCC-3′ R 5′-CACTTGTACACAGGAGGCTC-3′	122	61
	Vβ13-3	F 5′-AGCCCCAGTTCTAATTTACC-3′ R 5′-CTGACCAGCACTCACTTGCA-3′	122	61
	Vβ14	F 5′-GACTGATTCTCTGAAGGGACA-3′ R 5′-CAAGGAGACAGAACACTGCC-3′	155	61
RSS region	Vβ12-1	F 5′-CATGAGTGCCTTGGAACTGG-3′ R 5′-TTACAGAAAGCCAGTAGCTTTG-3′	87	62
	Vβ13-1	F 5′-TCAGGAAGTCCCTGCCTCTA-3′ R 5′-TGGCCTTCGGGCAGCTAGAAA-3′	86	62
	Vβ13-3	F 5′-CTAGCTCTCTGTGTACCCCA-3′ R 5′-AGGATGAGACTCATGCTGTG-3′	91	60
	Vβ14	F 5′-GGCCTAAAGGAACTAACTCCA-3′ R 5′-ACCATCAGCTTTGTGCACAG-3′	130	62

## Data Availability

The data presented in this study are available on request from the corresponding author.

## Supplementary Materials

The following supporting information can be downloaded at: https://www.mdpi.com/article/10.3390/ijms23137133/s1.
