# Supplementary material for: Chronic Hypergravity Induces a Modification of Histone H3 Lysine 27 Trimethylation at TCRβ Locus in Murine Thymocytes
Source: Int J Mol Sci. 2022 Jun 27;23(13):7133. doi: 10.3390/ijms23137133 (PMC9267123; doi:10.3390/ijms23137133)
Supplement: Supplementary file 1 [file ijms-23-07133-s001.zip › ijms-1778285-supplementary.pdf]

Figure S1

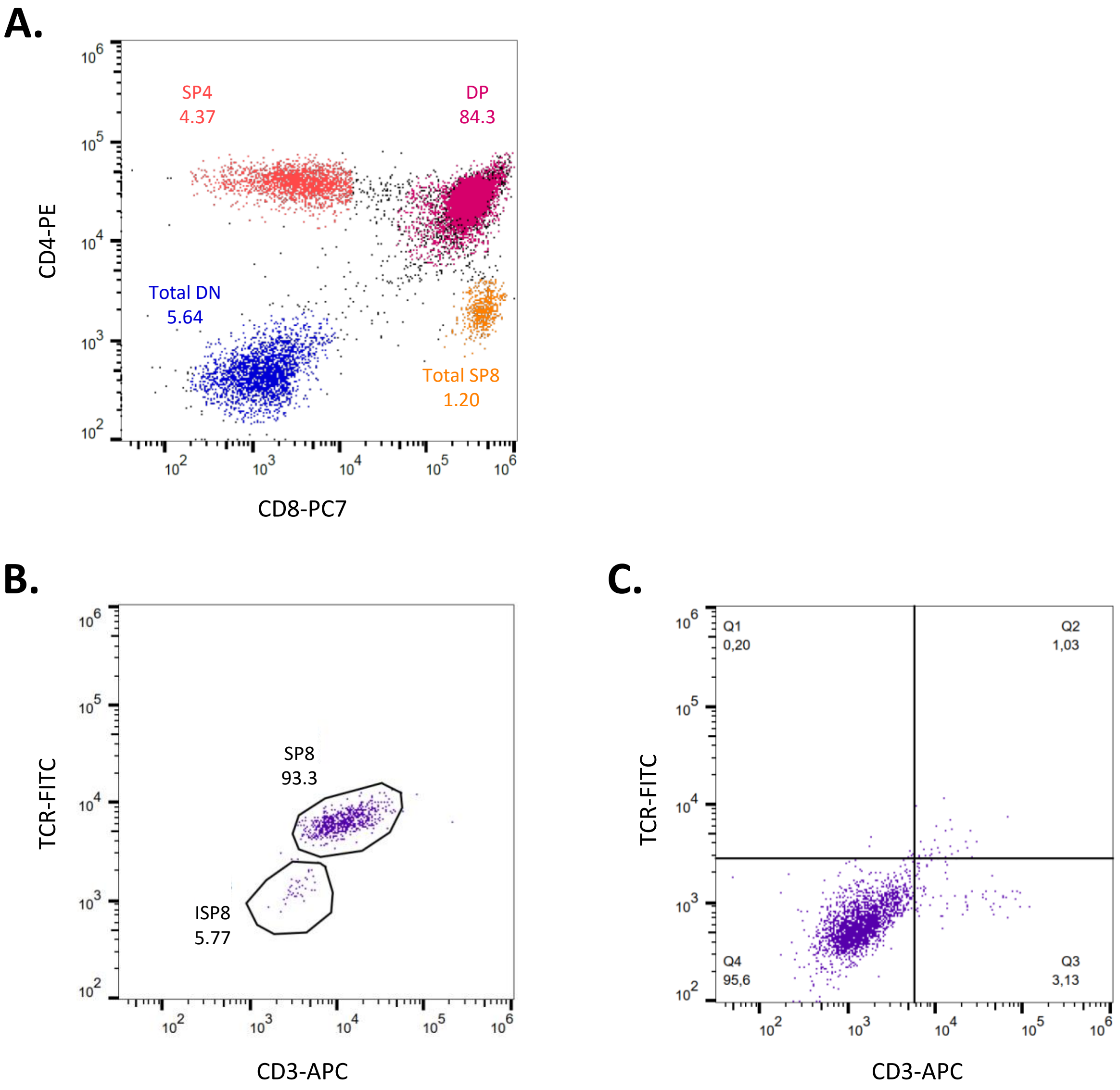

Supplementary Figure S1. *Gates designed to analyze thymocyte subpopulations in control (1G) and hypergravity (2G) mice.* Thymocytes were identified by flow cytometry. (A, B) Double positive (DP) and single positive (SP) subpopulations were identified using CD4, CD8, CD3 and TCR staining. (C) To ensure that DN are not contaminated by mature T-cells, we analyzed CD3 and TCR staining in this population.

Figure S2

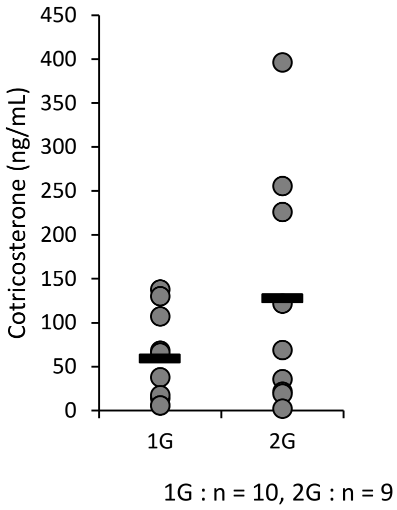

Supplementary Figure S2. *Serum corticosterone concentrations in 1G and 2G mice measured by ELISA*. Each dot corresponds to one mice. Mean is indicated as a black line. Mann-Whitney test was used to reveal statistically significant differences.

Figure S3

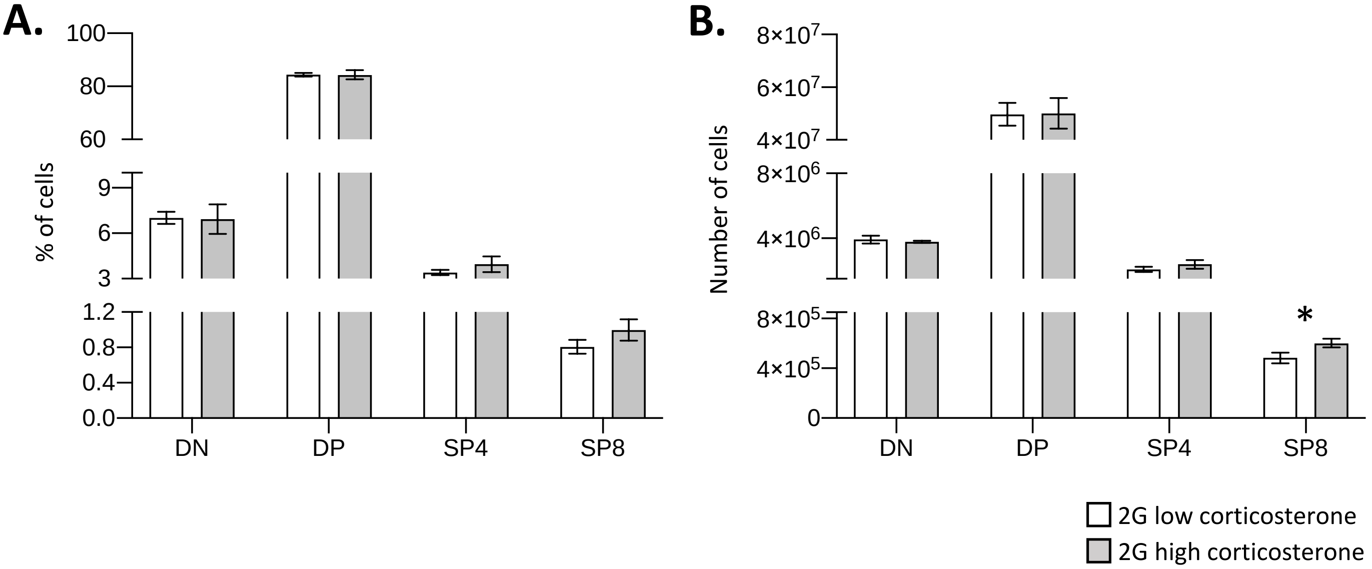

Supplementary Figure S3. *T lymphopoiesis is not affected by serum corticosterone concentration observed in 2G mice*. 2G mice were subdivided in two groups, one with normal serum corticosterone concentration (n=7) and one with higher serum corticosterone concentration (n=3). Analysis of T lymphopoiesis by flow cytometry. T-cell subpopulations were identified using CD4, CD8, CD3, TCR staining. For each mouse, staining was done in duplicate and mean was calculated. (A) Percentage of each subpopulation. (B) Absolute number of cells in each subpopulation. Data are mean ± SEM. t-tests were used to reveal statistically significant differences. * $p < 0.05$.

Figure S4

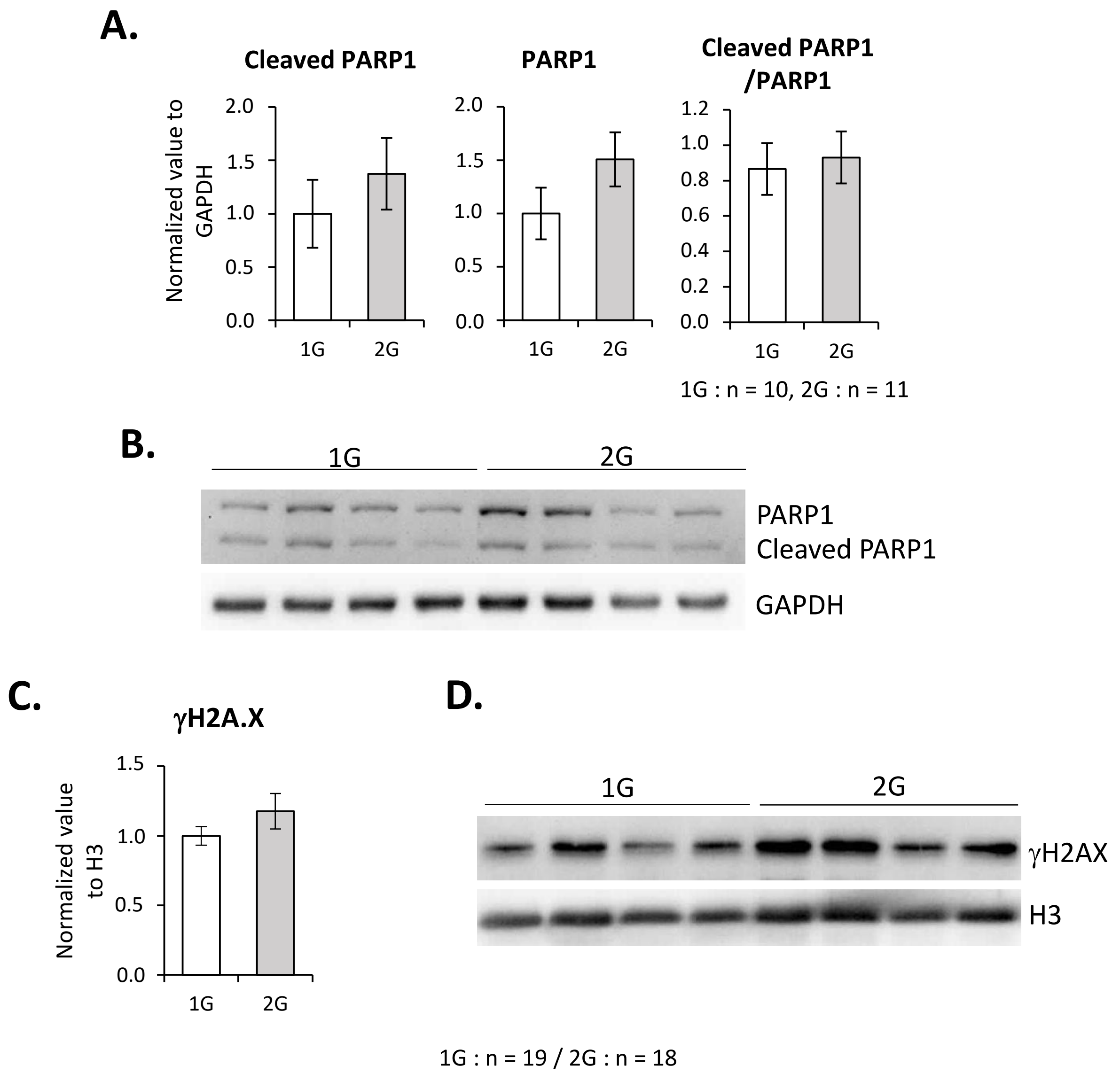

Supplementary Figure S4. *2G exposure does not affect PARP1 cleavage and γH2A.X level.* (A and B) Evaluation of thymocyte apoptosis *via* PARP1 cleavage detected by Western blotting. (A) Graphs presenting the quantification of cleaved PARP1 and PARP1 normalized to GAPDH. (B) Representative Western blot obtained with 4 mice in each group. (C and D) Evaluation of thymocyte DNA double strand break *via* γH2A.X level measured by Western blotting. (C) Graph presenting the quantification of γH2A.X normalized to H3. (D) Representative Western blot obtained with 4 mice in each group. Data are mean ± SEM. t-test or Mann-Whitney test did not reveal statistically significant differences.

Figure S5

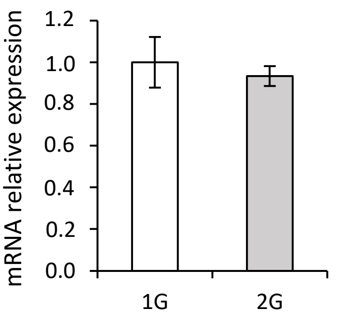

Supplementary Figure S5. *The transcription level of Cyclin D1 is not affected by hypergravity*. mRNA levels of Cyclin D1 were quantified using quantitative real-time PCR and normalized to three housekeeping transcripts (*Ppia*, *Eif3f*, *Rpl13a*). Data are the means ± SEM of 8 mice per group. t-test did not reveal a statistically significant difference.

Figure S6

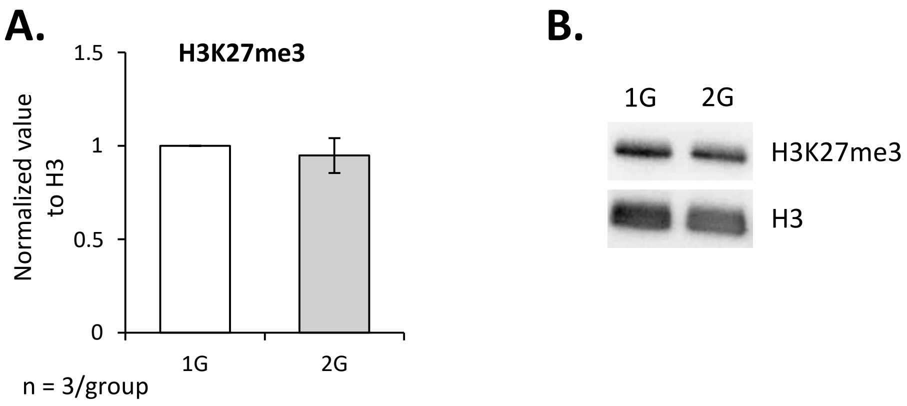

Supplementary Figure S6. *18h of 2xg hypergravity does not impact H3K27me3 level in the SCIET27 cell line.* H3K27me3 level was evaluated by Western blotting. (A) Graph presenting the quantification of H3K27me3 normalized to H3. (B) Representative Western blot. Data are mean ± SD. t-test was used to reveal statistically significant differences.

Figure S7

A.

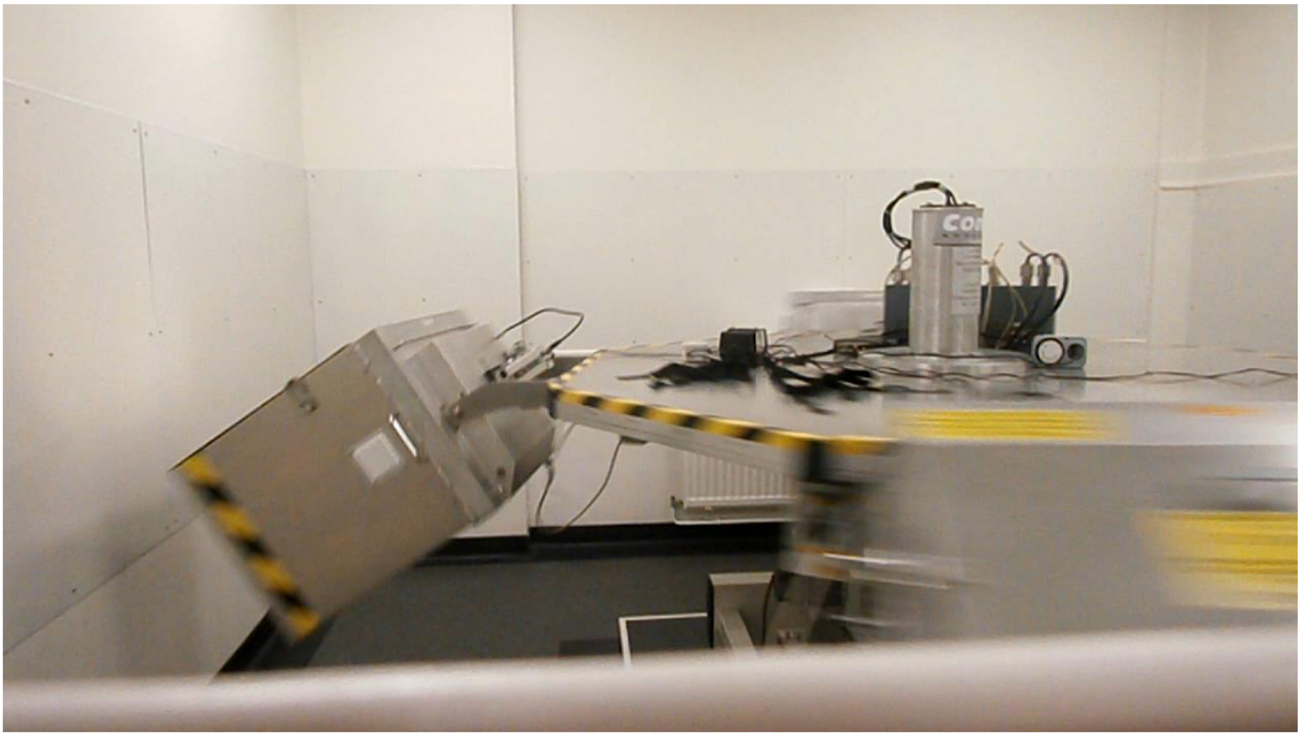

B.

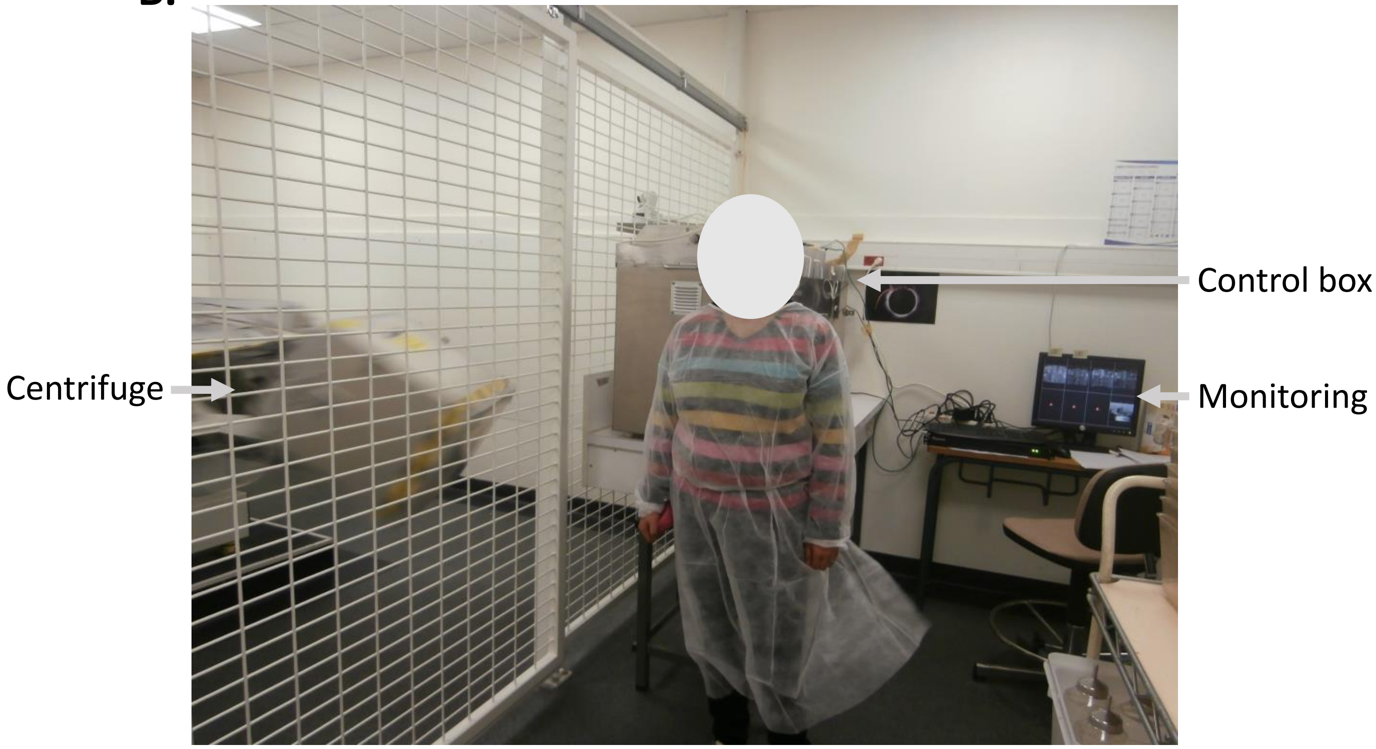

Supplementary Figure S7. *Mice hypergravity exposure*. The experimental setting includes the centrifuge (A), the control box (B) and the monitoring system (C).

Figure S8

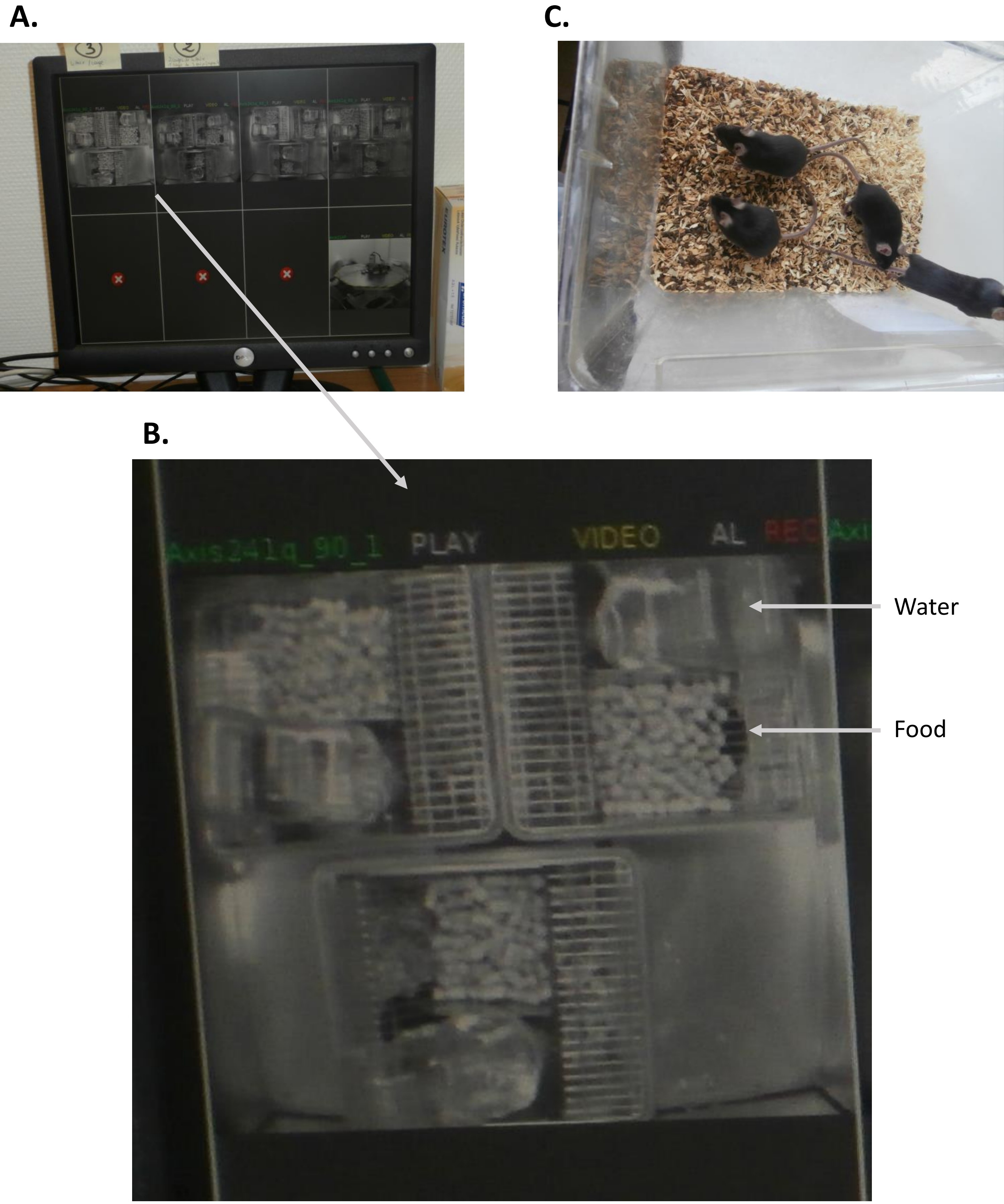

Supplementary Figure S8. *Mice monitoring during hypergravity exposure*. (A) Picture of the computer screen allowing mice monitoring during centrifugation via a camera placed in each gondola. Each rectangle corresponds to one gondola. (B) Zoom on a rectangle shown in A. Up to three cages containing food and water can be placed in a gondola. (C) Picture of mice, taken 5 minutes after the end of hypergravity exposure.

Figure S9

A.

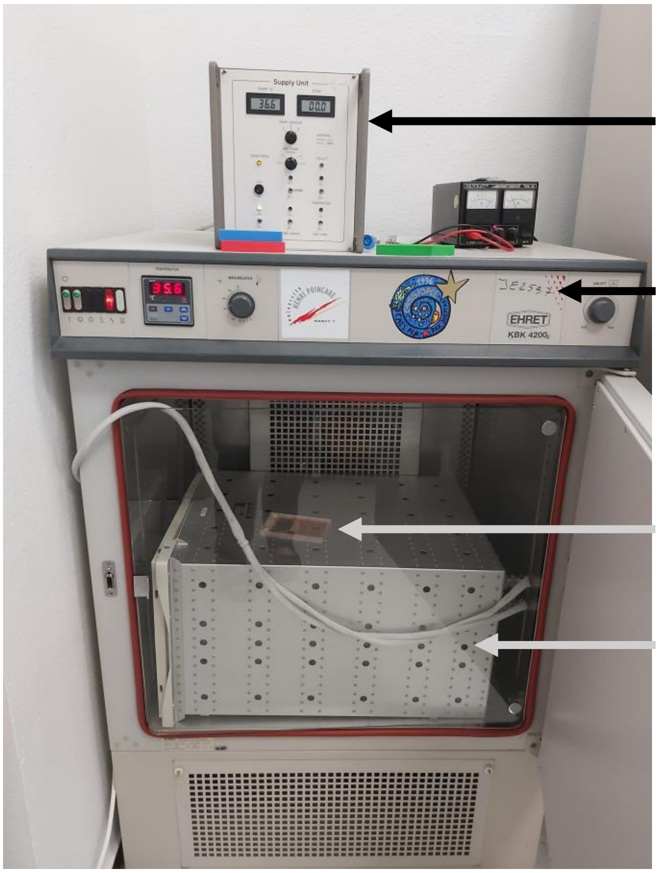

B.

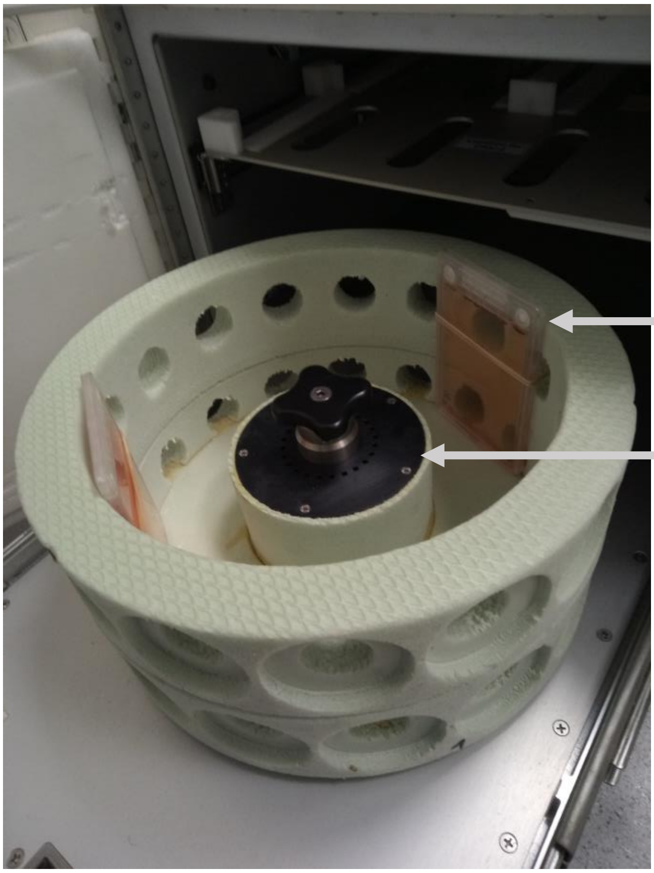

Supplementary Figure S9. *Hypergravity exposure of the SCIET27 cell line.* (A) Picture of the centrifuge placed in an incubator set at 37°C. A Petaka®G3 cell culture chamber was placed on the centrifuge as 1xg control. (B) Picture of rotor with two Petaka®G3 cell culture chambers.

Table S1. Antibodies, reagents and cell line used in this study.

Product	Target	Conjugate	Clone	Cat#	Supplier
Antibody	CD4	PE	RM4-5	100511	Biolegend (Ozyme, FR)
	CD8a	PECγ7	53-6.7	100721	
	TCRβ	FITC	H57-597	109205	
	CD3	APC	17A2	100235	
	GAPDH	-	-	G9545	Merck Millipore (FR)
	EZH2	-	-	07-689	
	EED	-	AA19	05-1320	
	H3K27me3	-	-	07-449	
	H3K4me2	-	-	07-030	
	H3K9ac	-	-	07-352	
	Suz12	-	D39F6	3737	Cell Signaling (USA)
	H3K9panmethyl	-	-	4069	
	γH2A.X	-	-	39118	Active Motif (BE)
	H3 C-terminal	-	-	39164	
	PARP1	-	-	GTX100573	Gentex (USA)
GSK126 inhibitor	EZH2	-	-	NDH49A	Interchim (FR)
SCIET27 cell line	SCIET27 is not a commercial cell line. It was kindly provided by I. Screpanti (Laboratory of Molecular Pathology, Sapienza University of Rome) and I. Aifantis (New York University, School of Medicine).				
